# Tick-Borne Relapsing Fever Borreliosis, Rural Senegal

**DOI:** 10.3201/eid1705.100573

**Published:** 2011-05

**Authors:** Philippe Parola, Georges Diatta, Cristina Socolovschi, Oleg Mediannikov, Adama Tall, Hubert Bassene, Jean François Trape, Didier Raoult

**Affiliations:** Author affiliations: Université de la Méditerranée, Marseille, France (P. Parola, G. Diatta, C. Socolovschi, O. Mediannikov, H. Bassene, J.F. Trape, D. Raoult);; Institut Pasteur de Dakar, Dakar, Senegal (A. Tall)

**Keywords:** Borrelia, Senegal, fever, borreliosis, Ornithodoros, ticks, vector-borne infections, bacteria, dispatch

## Abstract

Detecting spirochetes remains challenging in cases of African tick-borne relapsing fever. Using real-time PCR specific for the 16S rRNA *Borrelia* gene, we found 27 (13%) of 206 samples from febrile patients in rural Senegal to be positive, whereas thick blood smear examinations conducted at dispensaries identified only 4 (2%) as positive.

Tick-borne relapsing fever (TBRF), caused by several species of *Borrelia* spirochetes, is transmitted to humans through the bites of soft ticks of the genus *Ornithodoros* (through infected saliva or entry of infected coxal fluid at the bite site) (*1*). Wild rodents and insectivores are common reservoir hosts. TBRF-endemic foci persist around the world, where each *Borrelia* species causing relapsing fever appears to be specific to its tick vector. TBRF is responsible for recurring fever associated with spirochetemia. In recent years, the extent of relapsing fever caused by infection with *B. crocidurae*, transmitted by *O. sonrai* ticks and its effects on public health have only just begun to emerge. In Senegal, Mali, Mauritania, and the Gambia, where this tick is endemic, 2%–70% of animal burrows are inhabited by this tick vector, and an average of 31% of ticks are infected by *B. crocidurae* (*2**,**3*).

In Senegal, TBRF caused by *B. crocidurae* was recently determined to be the most common bacterial infection affecting the human population (*3*). A conventional diagnosis of TBRF is based on the detection of spirochetes in blood smears sampled during the acute febrile phase. However, TBRF is underdiagnosed in most disease-endemic areas, where blood smears are screened only for malaria parasites. Therefore, we used specific semiquantitative PCR to evaluate the role of TBRF as a cause of fever among malaria smear–negative patients in rural Senegal.

## The Study

During December 2008 through June 2009, we enrolled all patients with fever (axillary temperature >37.5°C) who had visited the dispensaries in Dielmo (13°43′N, 16°25′W; population 391) and Ndiop (14°33′N, 16°15′W; population 313) in Senegal (*3*). Informed consent was obtained from patients and from parents or legal guardians for children. Ethical clearance was granted by the national ethics committee of Senegal and the local ethics committee of Marseille, France.

If Giemsa-stained thick and thin blood smears were negative for malaria infection, then slides were checked again for *Borrelia* spp. A first screening was made at the dispensary and a second in Dakar by highly trained microscopists. Patients whose results were positive for *Borrelia* spp. received a 7-day regimen of oral doxycycline or erythromycin (for children <8 years of age and for pregnant women). In addition, 200 µL of whole blood was collected from each malaria-negative patient and used for DNA extraction with a QIAamp kit (QIAGEN, Hilden, Germany). Samples were washed and bound with QIAGEN columns and an adapted manual polyvinyl chloride pump (Fisher Scientific Inc., Strasbourg, France). The columns were stored at 4°C until final elution and PCRs were performed in Marseille.

*Borrelia* DNA was detected by using specific semiquantitative real-time PCR with primers (Bor16S3F, 5′**-**AGC CTT TAA AGC TTC GCT TGT AG-3′; Bor16S3R, 5′-GCC TCC CGT AGG AGT CTG G-3′), and a probe (Bor16S3P, 5′-6FAM- CCG GCC TGA GAG GGT GAA CGG-3′) that were designed for amplification of a 148-bp fragment of a 16S RNA–encoding gene. The specificities of the *Borrelia* spp. detection systems had been tested on DNA samples from 347 bacterial species, as described (*4*). All real-time PCRs were performed by using LightCycler 2.0 equipment and software (Roche Diagnostics GmbH, Mannheim, Germany). Appropriate handling and DNA extraction were controlled by qualitative PCR of the β-actin gene. Negative controls (sterile water and DNA from a sterile biopsy specimen) and positive controls (*B. crocidurae* DNA) were included for each test. All positive samples with a cycle threshold level of log-based fluorescence <36 (≈10–20 copies of spacer) were used to amplify the 148-bp gene fragments, which were subsequently sequenced, by PCR (*5*). Monthly variations were analyzed by making autocorrelation correlograms with PASW software version 17 (SPSS, Chicago, IL, USA).

A total of 134 patients were included in the study, and 206 samples were obtained. Several patients were seen multiple times with fever during the study period, and 1 sample was obtained during each independent febrile event. Test results for all controls were as expected. A total of 27 samples from 25 (13%) patients (17 male) were positive for *Borrelia* spp. by real-time PCR, including 26 (15.0%) of 172 from Dielmo and 1 (0.3%) of 34 from Ndiop. Most *Borrelia* spp.–positive patients (13; 56%) were <10 years of age, including 6 who were <5 years of age. A total of 8 (32%) patients were 5–10 years of age, and 3 were >20 years of age. Incidence rates in Dielmo were from 0.26 in November 2008, 0.51 in December 2008 and January 2009, 0.77 in March and May 2009, and 1.79 in June and July 2009.

Among the 27 samples positive by real-time PCR, only 4 (15%) had been identified as positive by thick smears at the dispensary, and only 15 (56%) had been identified as positive by highly trained microscopists who conducted a second thick-smear screening in Dakar. After PCR results were known, thick smears were examined again, and 3 more were found to be positive.

Two patients, 1 man and 1 woman, had *Borellia* spp.–positive results by real-time PCR and by thick smears when they were seen 2 times within 11 and 49 days, respectively. Among the group of 179 PCR-negative samples, no *Borrelia* spp. had been observed on thick smears. All DNA sequences obtained from these samples were identical and showed 100% identity with *B. crocidurae, B. duttonii*, *B. hispanica*, and *B. parkeri*.

## Conclusions

Our specific semiquantitative PCR results demonstrated that the sensitivity of thick blood smear analysis was dramatically low (15%) when performed in standard conditions in a dispensary and remained low (56%) even when performed by trained microscopists. Similar results have recently been shown for patients infected with *B. hispanica* in Morocco (*6*) or TBRF patients in Tanzania and Togo (*7**,**8*). In the study reported here, a limited quantity of DNA was available because other causes of fever were also screened (J.F. Trape, unpub. data). Therefore, we did not amplify and sequence a larger portion of the 16S RNA gene to definitively distinguish the *B. crocidurae* endemic to Senegal from other TBRF-causing borreliae.

This study highlights the endemicity of TBRF in this rural area of western Africa, where villagers are settled agricultural workers (Figure) (*3*). Rodent and insectivore burrows are found in almost all households; burrow openings were located inside the bedrooms of traditional huts built with mud and of houses with cement floors and walls. Transmission is mainly nocturnal. The bites are painless, and tick blood meals last from a few minutes to half an hour (*9*).

**Figure:**
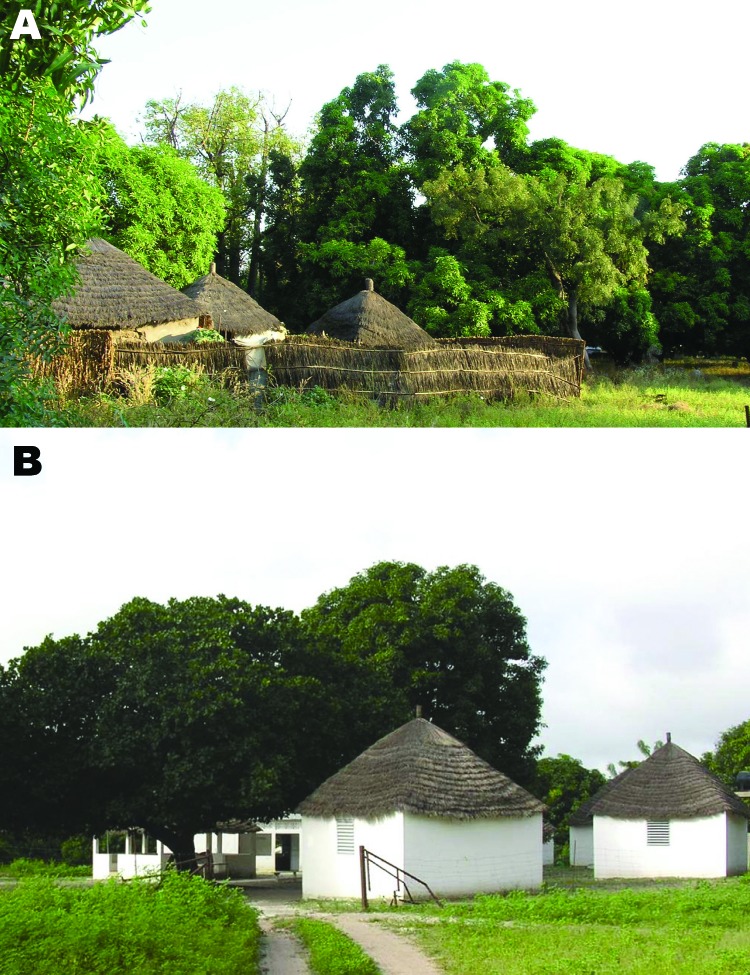
A) Dielmo village in Senegal. B) Health and clinical research station in Dielmo, where a longitudinal prospective study for long-term investigation of host–parasite associations has been conducted since 1990.

*B. crocidurae* accounts for high fever, frequent neurologic complications (*10*), and up to 9 recurrences over several months, but the mortality rates and early delivery by pregnant women caused by *B. crocidurae* seem to be lower than those caused by *B. duttonii* (*3*). In addition to relapses, repeat infections in the same person are common (*8*), as found in 2 patients reported here. Molecular methods showed the proportion of TBRF in febrile patients to be as high as 15% in Dielmo and incidence rates to be up to 1.79% per month in June and July of 2009. This finding confirms the increased incidence of TBRF that was noted between 1996 and 2002 (*11*).

Ideally, patients living in Senegal with unexplained fever should be tested for TBRF by molecular methods. However, this technique is more readily available to travelers returning from this country (*12*) than to its citizenry.
